# Artificial intelligence-based computer-aided detection systems for adenomas during colonoscopy: a systematic review and Bayesian network meta-analysis

**DOI:** 10.3389/frai.2026.1880524

**Published:** 2026-09-09

**Authors:** Zhenjia Fan, Danyan Li, Jixiang Liu, Yudi Zhuo, Shengsheng Zhang

**Affiliations:** Digestive Disease Center, Beijing Hospital of Traditional Chinese Medicine, Capital Medical University, Beijing, China

**Keywords:** adenoma detection rate, artificial intelligence, colonoscopy, computer aided detection, network meta-analysis

## Abstract

**Background and aims:**

Artificial intelligence-based computer-aided detection (CADe) systems have been developed to enhance the adenoma detection rate (ADR) during colonoscopy, but their performance is unknown. We primarily aimed to compare the effectiveness of each CADe system with conventional colonoscopy (CC). As a secondary objective, we performed an exploratory comparison among different CADe systems.

**Methods:**

A systematic literature search of 6 databases was conducted to find randomized controlled trials (RCTs) evaluating the use of CADe systems during colonoscopy. A Bayesian network meta-analysis was performed on the included studies using R 4.5.2 software. The primary outcome was ADR.

**Results:**

A total of 21 RCTs involving 19,006 participants were included. Nine CADe systems were compared. For ADR based on modified intention-to-treat (ADR-mITT), DEEP^2^ (RR 1.37; 95% CrI 1.07–1.76), Eagle-Eye (RR 1.19; 95% CrI 1.02–1.38), and ENDO-AID (RR 1.27; 95% CrI 1.13–1.41) were significantly higher than CC. For ADR based on intention-to-treat (ADR-ITT), AQCS was significantly higher than CC. For adenoma per colonoscopy (APC) and diminutive adenoma detection rate (DADR), ENDO-AID showed significant advantages over CC. However, no significant differences were observed among CADe systems in terms of high-risk lesion detection rate (including advanced adenoma detection rate [AADR] and sessile serrated lesion detection rate [SDR]) or withdrawal time (WT).

**Conclusion:**

Compared with CC, the CADe system significantly improved ADR. However, given the limited direct comparative evidence and the star-shaped network, the results, especially the differences among systems, should be interpreted cautiously.

**Systematic review registration:**

https://www.crd.york.ac.uk/PROSPERO/view/CRD420261290518, identifier: CRD420261290518.

## 1 Introduction

Colorectal cancer (CRC) ranked as the third most commonly diagnosed cancer and the second leading cause of cancer-related deaths, with over 1.14 million new cases and 538,000 deaths reported in 2022 (Foroutan et al., 2025; Neto et al., 2026). Adenomas are common precursors of CRC (Pan et al., 2026). Colonoscopy, as the gold standard for CRC screening and prevention, can effectively prevent the occurrence of CRC by detecting and removing adenomas during the procedure (Wang et al., 2025). The adenoma detection rate (ADR), defined as the percentage of colonoscopies in which one or more adenomas are identified, is closely associated with the incidence of post-colonoscopy CRC (Chang et al., 2026; Yabuuchi et al., 2025). A higher ADR is inversely associated with the CRC risk. The risk of developing CRC decreases by 3% when ADR increases by 1% (Corley et al., 2014; Kaminski et al., 2010). It is very important to improve the ADR for the prevention of CRC. However, the adenoma miss rate in conventional colonoscopy (CC) remains as high as 26%, posing a significant challenge in preventing CRC (Orzeszko et al., 2025). Therefore, enhancing the lesion detection during colonoscopy is the core challenge in improving CRC prevention and treatment.

In recent years, the advancement of artificial intelligence (AI) technology has introduced novel solutions to this clinical challenge (Xu et al., 2025; Xu et al., 2025). The AI-based computer-aided detection (CADe) system is designed to analyze endoscopic video streams in real time, automatically identify, and mark colorectal lesions to assist endoscopists in reducing the risk of missing lesions (Mănuc et al., 2025). Numerous trials have demonstrated that the CADe systems can significantly improve ADR, representing a new advancement in lesion screening.

At present, various CADe systems have been developed and applied in clinical or research settings worldwide, such as EndoScreener, DEEP^2^, CAD EYE, ENDO-AID, and GI-Genius (Spadaccini et al., 2025). These systems differ in their algorithmic architecture, training data, and methods of integration with endoscopic equipment (Zhu et al., 2025). Whether the performance of each CADe system differs by lesion types remains an open question.

The primary aim of this study was to compare the effectiveness of each CADe system with CC in improving ADR and other outcomes. These outcomes include ADR based on modified intention-to-treat (ADR-mITT), ADR based on intention-to-treat (ADR-ITT), adenoma per colonoscopy (APC), diminutive adenoma detection rate (DADR, ≤ 5 mm), advanced adenoma detection rate (AADR), sessile serrated lesion detection rate (SDR), and withdrawal time (WT). As a secondary aim, exploratory objective, we compared the relative performance among different CADe systems. Given limited data, differences among systems should be considered exploratory. We aimed to provide clinical summary estimates, rather than to establish definitive superiority among systems.

## 2 Methods

This study was performed following the Preferred Reporting Items for Systematic Reviews and Meta-analysis (PRISMA) guidelines (Page et al., 2021). The study protocol was registered in PROSPERO (CRD420261290518).

### 2.1 Search strategy

We conducted a comprehensive search of six databases (PubMed, Embase, MEDLINE, Scopus, Web of Science, CENTRAL) to identify all randomized controlled trials (RCTs) that had been published since the inception of the databases up to May 2025. Search terms included “Colon,” “Rectum,” “Adenoma,” “Computer Aided Detection”, “Artificial Intelligence”, “Randomized Controlled Trial”. The detailed search strategy is shown in Supplementary Table S1. Two reviewers independently conducted the literature search, with any different opinions resolved through consultation with the third reviewer.

### 2.2 Inclusion and exclusion criteria

Inclusion criteria: (1) Participants aged ≥18 years who needed colonoscopy; (2) Participants in the intervention group received colonoscopy with the assistance of the CADe system; (3) Participants in the control group received CC without the assistance of the CADe system; (4) Primary outcome (ADR) must be reported, and secondary outcomes including APC, DADR, AADR, SDR and WT may be reported or not; (5) The study type is a RCT.

Exclusion criteria: (1) Participants who had inflammatory bowel disease (IBD) or previous colonic surgery; (2) Extended functions of colonoscopy such as distal attachment and image enhancement devices were used during colonoscopy screening for polyps; (3) The name of the computer-aided detection system was unknown; (4) Studies with incomplete data or unavailable full texts; (5) Studies with fewer than 30 participants in any group; (6) Studies that were case reports, letters, reviews, protocols, animal or cell experiments. (7) Duplicate publications.

Extended functions of colonoscopy have been shown to independently increase ADR. When these techniques are used together with CADe systems, it becomes difficult to separate the effect attributable to CADe from that of the co-administered intervention. To estimate the effectiveness of CADe with minimal confounding, we excluded trials that combined CADe with such extended functions.

Two reviewers independently screened the literature, with any different opinions resolved through consultation with the third reviewer.

### 2.3 Data extraction

Two reviewers independently extracted the following data from the included studies: the first author, year of publication, country, the date of trials, sex, age, intervention method, sample size, outcomes, funding. When the study was a crossover trial, only the data of the first colonoscopy inspection were extracted. Any different opinions were resolved through consultation with the third reviewer.

### 2.4 Study quality assessment

The risk of bias was evaluated using Review Manager 5.3 based on the Cochrane Risk-of-Bias Tool for Randomized Trials. The quality of evidence was evaluated using the Grading of Recommendations Assessment, Development, and Evaluation (GRADE) approach (Brignardello-Petersen et al., 2018). Given that there were numerous CADe systems on the network, we adopted a focused comparative approach in the GRADE assessment (Chen et al., 2025). Specifically, we created evidence profiles comparing each CADe system with CC.

Two reviewers independently assessed the quality of the included studies, with any different opinions resolved through consultation with the third reviewer.

### 2.5 Statistical analysis

We conducted a Bayesian network meta-analysis (NMA) of the included RCTs using the gemtc and rjags packages in R 4.5.2 software. The Markov Chain Monte Carlo (MCMC) method was used to fit the models (Souza et al., 2026). The Brooks-Gelman-Rubin method was used to assess model convergence. Values of the Potential Scale Reduction Factor (PSRF) closer to 1 indicate better convergence (Xiong et al., 2024). We properly adjusted the sample iteration and number of burn-in. The random-effects models were used to conduct NMA (Song et al., 2026). The consistency model was adopted for analysis since the network was star-shaped without closed loops (Huang et al., 2024). The pooled results were expressed as risk ratios (RR) for dichotomous outcomes, and mean differences (MD) for continuous outcomes, all reported with 95% credible intervals (CrI) (Terasawa et al., 2025). The primary result analysis was based on intention-to-treat (ITT) and modified intention-to-treat (mITT) (participants with inadequate bowel preparation and unsuccessful intubation were excluded from the mITT analysis), while the secondary results were analyzed based on mITT. When studies reported Mean and 95% CI without SD, the SD was calculated based on the methods described in the Cochrane Handbook (Higgins et al., 2024). When outcomes were reported as Median and IQR, the Mean and SD were calculated if the data showed no skewness; otherwise, the data were not included in the meta-analysis (Zaaroura et al., 2023). Cochran’s *Q* test and the *I*^2^ statistic were used to assess heterogeneity, with *p* < 0.1 and *I*^2^ > 50% indicating substantial heterogeneity (Ye and Huang, 2025). Transitivity assessment was performed for potential effect modifiers (population characteristics, indications, endoscopist experience, baseline ADR, bowel preparation, and study center type). A sensitivity analysis including all trials that used extended functions of colonoscopy was conducted to evaluate the stability of the primary outcome. The Egger test was used to determine whether publication bias existed in this NMA (Ahmed et al., 2025).

## 3 Results

### 3.1 Study selection and characteristics

**Figure 1:**
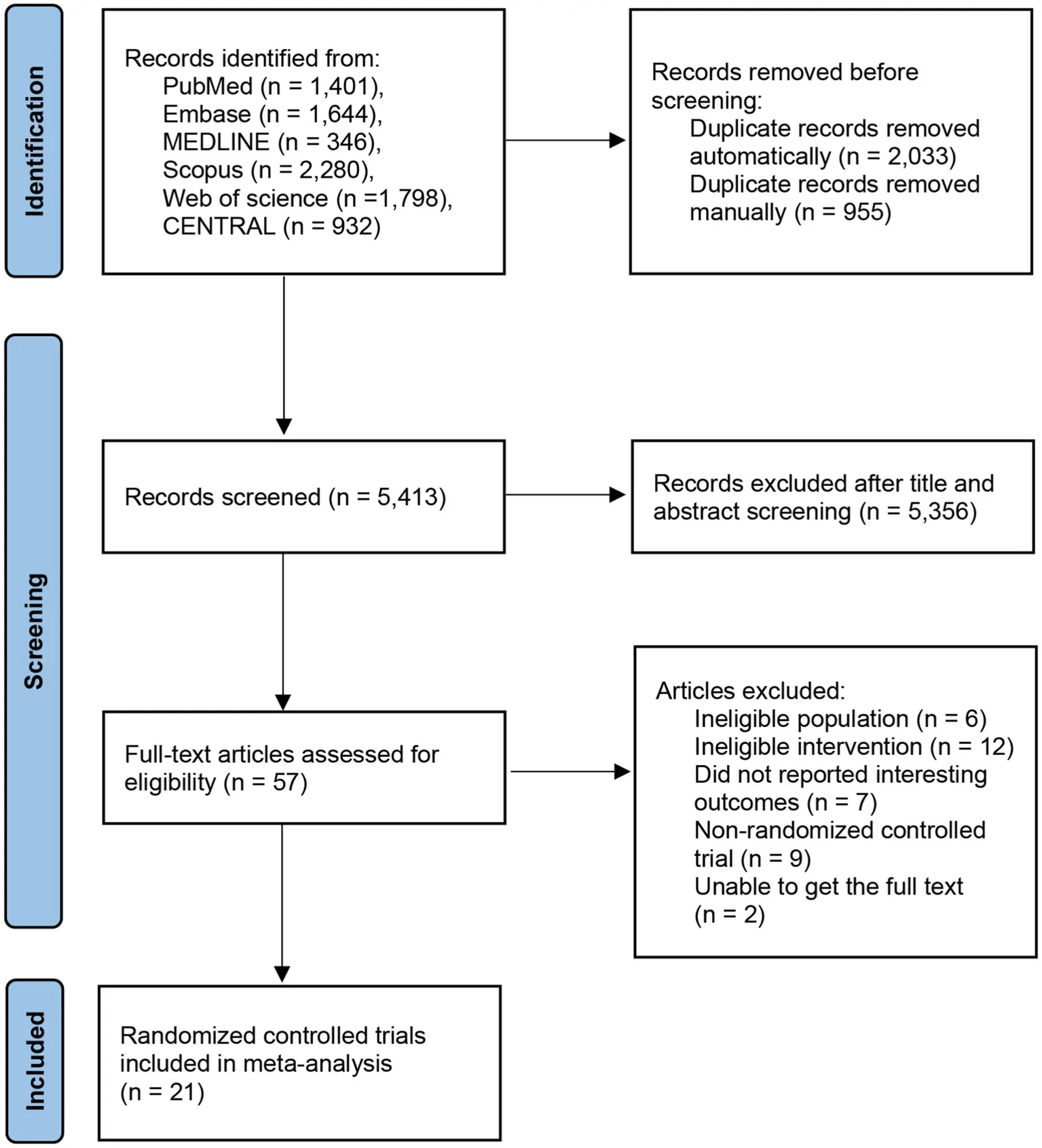
Flow diagram of the study screening process.

A total of 8,401 records were identified through the literature search. After removing duplicates, 5,413 articles remained for title and abstract screening. We then conducted a full-text screening of 57 articles. 36 of them were excluded, and finally 21 RCTs were included in the NMA (Figure 1).

Among the 21 included studies, there were a total of 19,006 participants, comprising 9,523 in the control group and 9,483 in the intervention group (Alali et al., 2025; Desai et al., 2024; Gimeno-García et al., 2022; Hiratsuka et al., 2025; Karsenti et al., 2023; Lachter et al., 2023; Lau et al., 2024; Liu et al., 2020; Liu et al., 2025; Mangas-Sanjuan et al., 2023; Nakashima et al., 2023; Repici et al., 2022; Spada et al., 2025; Wallace et al., 2022; Wang et al., 2020a; Wang et al., 2020b; Wei et al., 2023a; Wei et al., 2023b; Xu et al., 2023; Yamaguchi et al., 2024; Yao et al., 2022). This NMA covers 10 countries: 7 in China, 3 in USA, 3 in Japan, 2 in Spain, 1 each in Italy, Israel, Kuwait and France, and 2 in multiple countries. Among the included studies, 5 were funded by industry, 4 received equipment support from industry, 1 was funded by government, 1 was funded by a non-profit organization, 5 were unfunded, and 5 did not report funding sources. All included trials, conducted between September 2018 and April 2023, were analyzed. Detailed information is shown in Table 1.

**Table 1:** Characteristics of the included trials.

Author and Year	Country	Date of Trials	Sex (CADe/CC), % Male	Age (CADe/CC), Mean (SD) or Median [IQR]	Intervention Method	Sample Size (CADe/CC)	Outcomes	Funding
Alali et al., 2025	Kuwait	Sep. 2022–Mar. 2023	58.8/41.2	51.1 (7.7)/54.5 (8.3)	CAD EYE	51/51	ADR-mITT, APC, WT	NO
Desai et al., 2024	United States	NA	51.5/48.3	58.9 (9.5)/59.3 (10.1)	CAD EYE	509/522	ADR-mITT, APC, DADR, AADR, SDR, WT	Industry
Gimeno-García et al., 2022	Spain	Nov. 2021–Jan. 2022	52.9/52.9	62.99 (10.26)/64.71 (11.79)	ENDO-AID	mITT: 155/157; ITT: 185/185	ADR-mITT, ADR-ITT, APC, AADR, WT	Industry for equipment
Hiratsuka et al., 2025	Japan	Feb. 2022–Nov. 2022	64.58/63.04	67.4 (3.6)/70.7 (2.6)	CAD EYE	48/46	ADR-mITT, APC	NA
Karsenti et al., 2023	France	May 2021–May 2022	48.0/49.2	58.4 (11.4)/58.4 (11.8)	GI-Genius	1003/1012	ADR-mITT, APC, AADR	NO
Lachter et al., 2023	Israel	Mar. 2021–Dec. 2021	47	62.4 (10.29)	DEEP^2^	330/344	ADR-mITT, DADR, AADR, WT	Industry
Lau et al., 2024	China	Apr. 2019–Jul. 2022	53.1/55.5	66.00 (10.05)/65.36 (11.33)	ENDO-AID	386/380	ADR-mITT, APC, AADR, SDR, WT	Industry for equipment
Liu et al., 2020	China	Sep. 2018–Feb. 2019	45.80/48.87	49.84 (13.11)/48.79 (13.00)	EndoScreener	393/397	ADR-ITT	NO
Liu et al., 2025	China	Aug. 2021–Sep. 2022	51.4/56.1	NA	AQCS	627/627	ADR-ITT	NA
Mangas-Sanjuan et al., 2023	Spain	Apr. 2021–Mar. 2022	53.7/53.2	61 (6)	GI-Genius	1608/1596	ADR-mITT, APC, DADR, AADR, WT	Industry for equipment
Nakashima et al., 2023	Japan	Jan. 2021–Mar. 2022	73.9/69/7	54.9 (10.9)/55.9 (10.4)	CAD EYE	207/208	ADR-ITT	NO
Repici et al., 2022	Italy, Switzerland	Feb. 2020–Dec. 2020	52.7/47.3	61.9 (9.8)/62.6 (10.2)	GI-Genius	330/330	ADR-ITT	Industry for equipment
Spada et al., 2025	Italy	Oct. 2021–Mar. 2023	50.9/47.1	mITT: 62.3 (10.5)/61.9 (10.0); ITT: 62.3 (10.5)/61.9 (9.96)	ENDO-AID	mITT: 578/580; ITT: 611/617	ADR-mITT, ADR-ITT, APC, AADR, SDR, WT	NA
Wallace et al., 2022	Italy, United Kingdom, United States	Feb. 2020–May 2021	68.97/67.54	63.0 (8.2)/64.6 (8.1)	GI-Genius	116/114	ADR-mITT, APC	Industry
Wang et al., 2020a	China	Jun. 2019–Sep. 2019	50.54/46.49	47.72 (10.82)/47.19 (10.38)	EndoScreener	184/185	ADR-mITT, APC, WT	NA
Wang et al., 2020b	China	Sep. 2018–Jan. 2019	50/53	49.0 [39.0–60.0]/49.0 [40.3–56.0]	EndoScreener	484/478	ADR-ITT	NO
Wei et al., 2023a	United States	Sep. 2020–Sep. 2021	50.8/51.2	58.0 (7.9)/57.6 (7.9)	EndoVigilant	387/382	ADR-mITT, APC, SDR, WT	Industry
Wei et al., 2023b	United States	Sep. 2020–Dec. 2021	95.2/97.5	68.7 (7.5)/67.8 (8.0)	EndoVigilant	124/120	ADR-ITT	Industry
Xu et al., 2023	China	Nov. 2019–Aug. 2021	46.5/47.3	57.49 (7.55)/57.03 (7.43)	Eagle-Eye	mITT: 1238/1289; ITT: 1519/1540	ADR-mITT, ADR-ITT, APC, DADR, AADR, SDR	NA
Yamaguchi et al., 2024	Japan	May 2021–Mar. 2022	51.3/55.9	63.1 (10.8)/63.3 (11.8)	CAD EYE	113/118	ADR-mITT, APC, SDR, WT	Non-profit
Yao et al., 2022	China	Jul. 2020–Oct. 2020	45.15/42.07	50.69 (13.15)/50.85 (13.56)	EndoAngel	268/271	ADR-mITT, APC, DADR, AADR, SDR, WT	Government

### 3.2 Assessment of model convergence

The number of iterations and burn-in for each outcome is detailed in Supplementary Table S2. Model convergence was assessed using the Brooks-Gelman-Rubin diagnostic method. The results showed that the PSRF values for all parameters ranged from 1.00 to 1.01, indicating that the Markov chains had converged (Supplementary Figure S1).

### 3.3 Network geometry

A total of 15 trials contributed to the network for ADR-mITT, 9 trials for ADR-ITT, 14 trials for APC, 9 trials for AADR, 10 trials for SDR, 11 trials for WT. Network plots were shown in Supplementary Figure S2.

### 3.4 Risk of bias assessment

**Figure 2:**
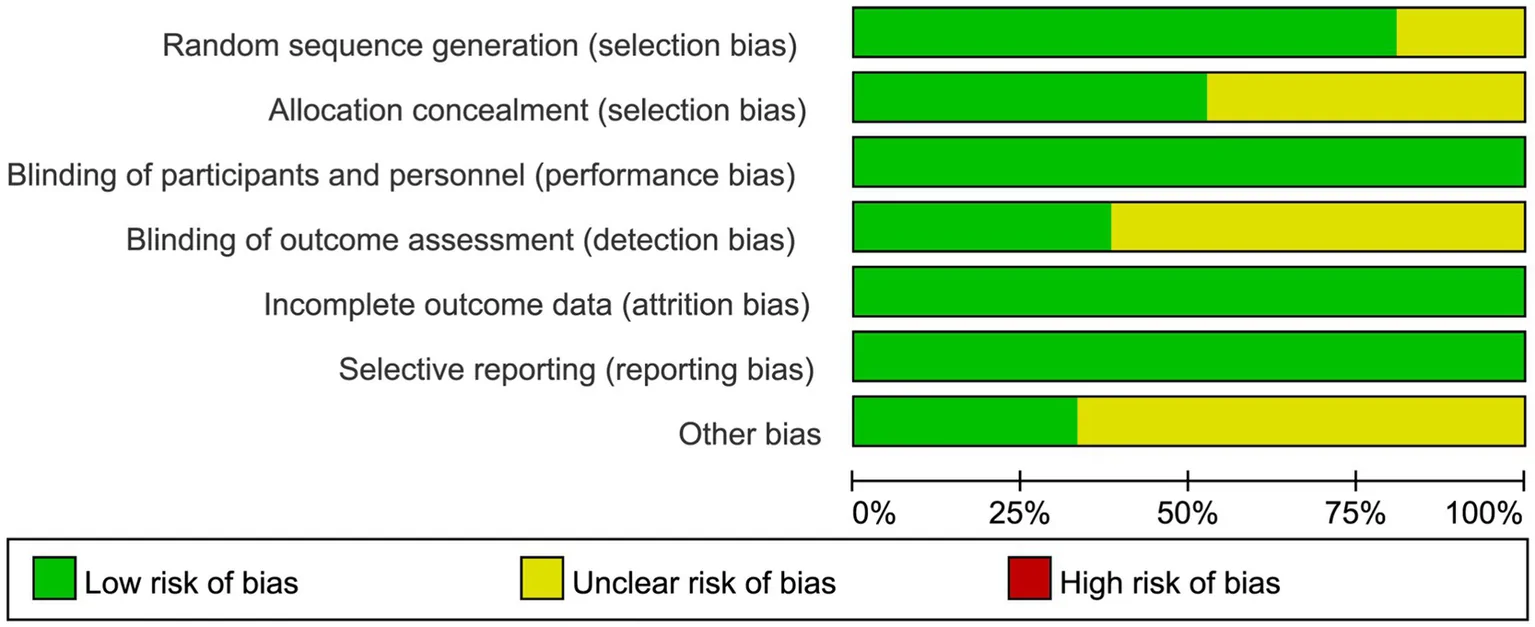
Risk of bias graph.

Overall, the results of the assessment indicate that the included trials have a low risk of bias in most domains. Some trials had inadequate allocation concealment descriptions and insufficient blinding. No serious issues of missing outcome data or selective reporting were noted. Detailed information is shown in Figure 2 and Supplementary Figure S3.

### 3.5 Outcomes

#### 3.5.1 ADR

**Figure 3:**
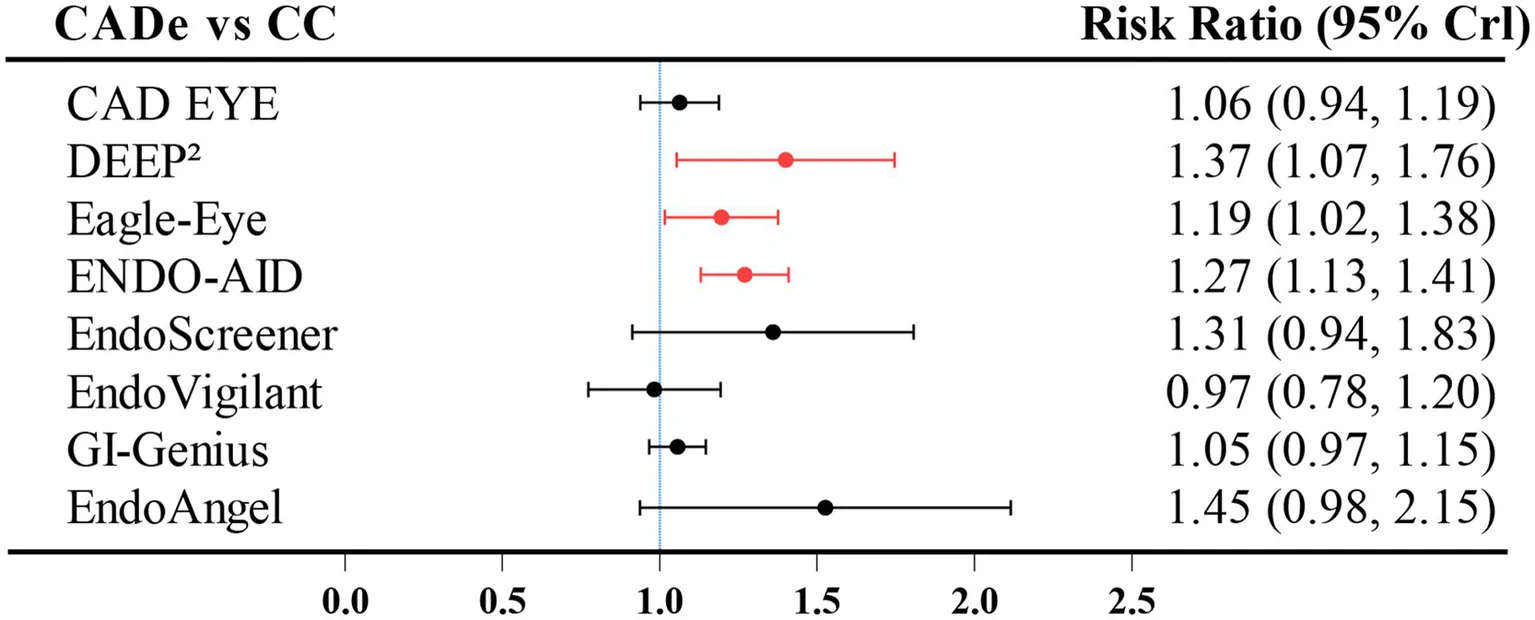
Forest plot of the comparisons between CADe systems and CC for ADR-mITT.

ADR-mITT 15 RCTs reported ADR based on mITT, involving 14,021 participants. The NMA results indicate that the ADR for DEEP^2^ (RR 1.37; 95% CrI 1.07–1.76), Eagle-Eye (RR 1.19; 95% CrI 1.02–1.38), ENDO-AID (RR 1.27; 95% CrI 1.13–1.41) was significantly higher than CC (Figure 3; Supplementary Figure S4A). No significant heterogeneity was observed (Supplementary Table S3).

**Figure 4:**
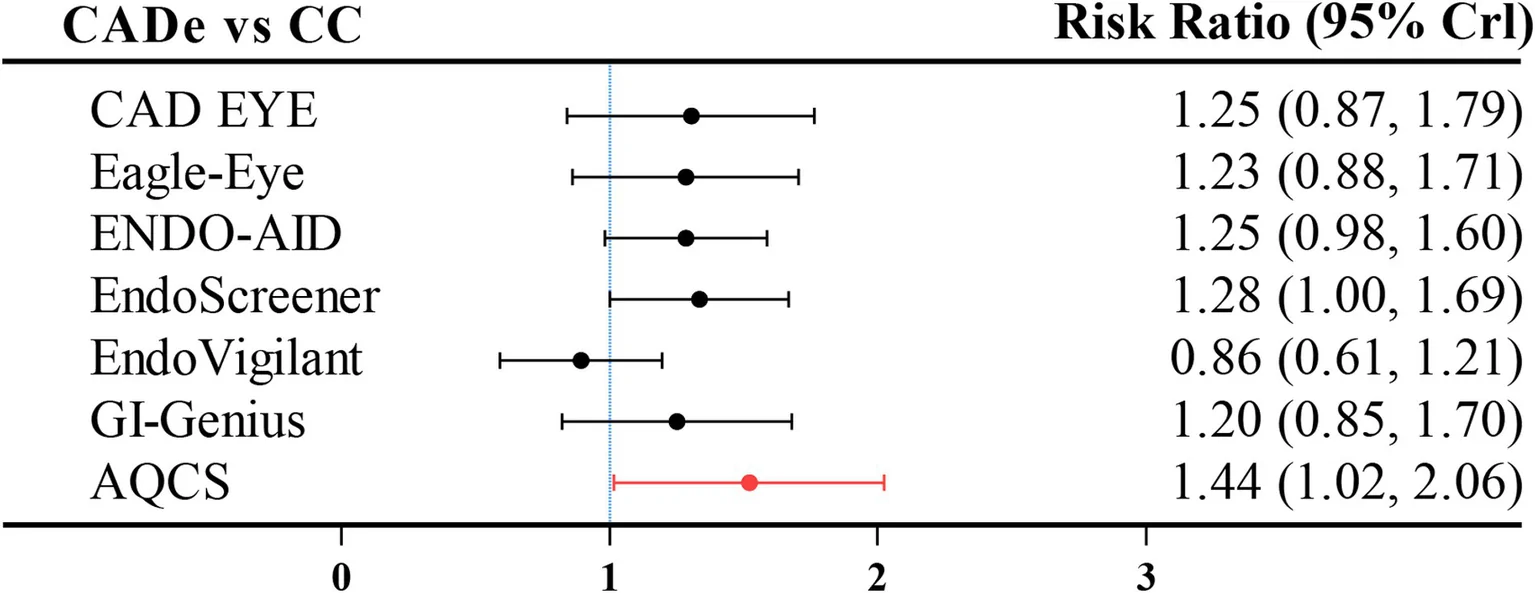
Forest plot of the comparisons between CADe systems and CC for ADR-ITT.

ADR-ITT 9 RCTs reported ADR based on ITT, involving 8,982 participants. The NMA results indicate that the ADR for AQCS was significantly higher than CC (RR 1.44; 95% CrI 1.02–2.06) (Figure 4, Supplementary Figure S4B). No significant heterogeneity was observed (Supplementary Table S4).

#### 3.5.2 APC

14 RCTs reported the results of APC, involving 13,347 participants. The NMA results indicate that the APC for ENDO-AID was significantly higher than CC (MD 1.53; 95% CrI 1.03–2.30) (Supplementary Figure S4C). No significant heterogeneity was observed (Supplementary Table S5).

#### 3.5.3 DADR

7 RCTs reported the results of DADR, involving 9,052 participants. The NMA results indicate that the DADR for ENDO-AID was significantly higher than CC (RR 1.53; 95% CrI 1.03–2.25) (Supplementary Figure S4D). No significant heterogeneity was observed (Supplementary Table S6).

#### 3.5.4 AADR

9 RCTs reported the results of AADR, involving 12,226 participants. The NMA results showed that compared with CC, CAD EYE (RR 1.02; 95% CrI 0.55–1.92), DEEP^2^ (RR 1.52; 95% CrI 0.68–3.44), Eagle-Eye (RR 1.31; 95% CrI 0.78–2.21), ENDO-AID (RR 1.01; 95% CrI 0.70–1.45), and GI-Genius (RR 1.04; 95% CrI 0.77–1.50) had higher AADR, but the differences were not statistically significant (Supplementary Figure S4E). Low heterogeneity was observed among the included studies (*p* = 0.2784, *I*^2^ = 20.7%) (Supplementary Table S7).

#### 3.5.5 SDR

7 RCTs reported the results of SDR, involving 7,021 participants. The NMA results showed that compared with CC, CAD EYE (RR 1.05; 95% CrI 0.28–2.39), ENDO-AID (RR 1.11; 95% CrI 0.43–2.87), and EndoVigilant (RR 1.03; 95% CrI 0.28–3.76) had higher SDR, but the differences were not statistically significant (Supplementary Figure S4F). Low heterogeneity was observed among the included studies (*p* = 0.3668, *I*^2^ = 25.0%) (Supplementary Table S8).

#### 3.5.6 WT

11 RCTs reported the results of WT, involving 9,159 participants. The NMA results showed that the WT of CAD EYE (MD 0.40; 95% CrI −0.29–1.04), DEEP^2^ (MD 0.60; 95% CrI −0.45–1.64), ENDO-AID (MD 0.18; 95% CrI −0.34–0.94), EndoScreener (MD 0.04; 95% CrI −0.95–1.03), EndoVigilant (MD 0.30; 95% CrI −0.69–1.29), GI-Genius (MD 0.80; 95% CrI −0.20−1.79), EndoAngel (MD 0.58; 95% CrI −0.55–1.70) were longer than that of CC, but the differences were not statistically significant (Supplementary Figure S4G). Low heterogeneity was observed among the included studies (*p* = 0.2992, *I*^2^ = 25.0%) (Supplementary Table S9).

### 3.6 Publication bias

We assessed publication bias for the 15 studies that reported ADR-mITT using Egger’s test (based on log RR). The result (*p* = 0.085) was above the 0.05 significance level, indicating no significant publication bias for this outcome (Supplementary Figure S5). The assessment was not performed for ADR-ITT due to the limited number of available studies.

### 3.7 Transitivity assessment

We assessed the transitivity of effect modifiers that may influence the indirect comparison results. Regarding bowel preparation quality, the mean BBPS scores across the included studies ranged from 7 to 8.35, with no obvious abnormal trend. The mean proportion of experienced endoscopists across the different CADe systems ranged from 50 to 100%, with no obvious abnormal trend. In addition, population characteristics, indication, baseline ADR, and study center type showed no obvious imbalances in most of the included trials (Supplementary Figure S6). However, residual confounding still cannot be excluded, the results of the indirect comparisons require cautious interpretation.

### 3.8 Sensitivity analysis

We performed a sensitivity analysis by including trials that used extended functions of colonoscopy Gong et al., 2020; Vilkoite et al., 2023). The results were not consistent in terms of effect magnitude. For example, for the EndoAngel system, the primary outcome risk ratio changed from 1.44 to 1.68 (Supplementary Table S10). This indicates that the use of extended colonoscopy functions may confound the estimation of the effect of CADe systems, supporting the decision to exclude them from the analysis.

### 3.9 Quality of evidence assessment

We used the GRADEpro software to create summary tables for the quality of evidence assessment results of ADR-mITT (Supplementary Table S11) and ADR-ITT (Supplementary Table S12). The tables show the certainty ratings (high, medium, low, or very low) for key comparisons.

## 4 Discussion

This Bayesian NMA included 21 RCTs involving 19,006 participants. We conducted a comparison of the performance between various CADe systems and CC. Results indicate that CADe colonoscopy demonstrated a significant and consistent advantage over CC in terms of ADR. The ADR-mITT of the DEEP^2^, ENDO-AID, and Eagle-Eye systems was significantly higher than that of CC. The ADR-ITT of the AQCS was significantly higher than CC. Additionally, ENDO-AID was significantly higher than CC in terms of APC and DADR. However, in terms of the detection rates of high-risk lesions (AADR and SDR), although most CADe systems showed increased detection rates, none of them reached statistical significance compared with CC. Besides, it is noteworthy that different systems showed a tendency to prolong WT, though none reached statistical significance compared with CC. These findings strengthen the evidence that the CADe system is an effective tool for improving colonoscopy quality.

Detailed technical specifications of each system are presented in Table S13. The included CADe systems vary in algorithmic architecture, training data, latency, and alert mechanisms. However, the associations between these technical specifications and the clinical outcomes observed in our NMA remain insufficiently quantified. Therefore, any observed differences in detection outcomes cannot be attributed to specific design features. Future studies should systematically report system version, training data sources, false-positive rates, latency, alert fatigue, endoscopist experience, and learning curves to enable meaningful comparisons across systems.

This NMA has some advantages compared with previous studies (Kumar et al., 2025). First, we analyzed more CADe systems such as DEEP^2^, Eagle-Eye, AQCS, and EndoVigilant. Second, our analysis encompassed a broader range of outcomes (ADR, APC, DADR, AADR, SDR, WT) to ensure a more comprehensive assessment of CADe systems. Third, we conducted separate analysis based on mITT and ITT, which is one of the key factors influencing the results.

However, this NMA also has certain limitations. First, although a large number of studies on CADe systems have been published, the number of studies we included is limited because we excluded studies that involved either extended colonoscopy functions or participants who had IBD or previous colonic surgery. Therefore, our conclusions are mainly applicable to CADe-assisted colonoscopy without the combination of other enhancement technologies, and to participants without IBD or previous colonic surgery. The performance of CADe systems combined with such techniques, as well as their performance in participants with IBD or previous colonic surgery, requires further investigation. Second, the limited number of available trials for certain outcomes (e.g., AADR, SDR) may affect the stability of the conclusions. Third, the network structure was star-shaped, with most studies comparing a single CADe system against CC. Direct comparisons between different CADe systems were sparse, which limits the strength of the relative differences. Although a transitivity assessment did not reveal gross imbalances, potential confounding cannot be ruled out. Therefore, the differences among systems should be viewed as exploratory and not as evidence of definitive superiority. Finally, some CADe systems received funding or equipment support from enterprises. Although randomized designs were employed in those trials, potential biases still require consideration.

In conclusion, this NMA confirms the overall effectiveness of CADe systems in improving colonoscopy ADR. For outcomes such as AADR, SDR, and WT, no significant differences were observed between CADe and CC. The exploratory differences among systems should be interpreted cautiously and the present analysis does not support conclusions about the superiority of any single system, given the limited direct comparative evidence and the predominantly star-shaped network. Future studies should focus on conducting more head-to-head comparative trials, evaluating the long-term impact of CADe systems on lesion detection, and establishing internationally unified standards for AI system performance evaluation and reporting, thereby further promoting the development of precision prevention for colorectal cancer.

## Data Availability

The original contributions presented in the study are included in the article/Supplementary material, further inquiries can be directed to the corresponding author.
