# Supplementary material for: Artificial intelligence-based computer-aided detection systems for adenomas during colonoscopy: a systematic review and Bayesian network meta-analysis
Source: Front Artif Intell. 2026 Sep 9;9:1880524. doi: 10.3389/frai.2026.1880524 (PMC13597736; doi:10.3389/frai.2026.1880524)
Supplement: Supplementary file 2 [file Table_1.doc]

**Supplementary Material**

**Table S1.** Search strategy of the study.

**Table S2.** Details of the Network Meta-analysis Models.

**Table S3.** The heterogeneity of the studies on ADR based on modified intention-to-treat.

**Table S4.** The heterogeneity of the studies on ADR based on intention-to-treat.

**Table S5.** The heterogeneity of the studies on APC.

**Table S6.** The heterogeneity of the studies on DADR based on intention-to-treat.

**Table S7.** The heterogeneity of the studies on AADR.

**Table S8.** The heterogeneity of the studies on SDR.

**Table S9.** The heterogeneity of the studies on WT.

**Table S10.** Effect estimates for CADe system compared with conventional colonoscopy.

**Table S11.** GRADE assessment results for ADR based on modified intention-to-treat.

**Table S12.** GRADE assessment results for ADR based on intention-to-treat.

**Table S13**. Key features of CADe systems.

**Figure S1.** Assessment of the model convergence for ADR based on modified intention-to-treat (Brooks-Gelman-Rubin plots).

**Figure S2.** The networks of included randomized controlled trials.

**Figure S3.** Risk of bias summary.

**Figure S4.** Comprehensive estimate for the comparison of outcomes in the network meta-analysis.

**Figure S5.** Egger’s test for the included studies involving ADR based on modified intention-to-treat.

**Figure S6.** Assessment of the transitivity.

**Table S1.** Search strategy of the study.

|  | **Search number** | **Search query** |
| --- | --- | --- |
| **PubMed** | #1 | ((((((("Adenoma"[Mesh]) OR ("Polyps"[Mesh])) OR ("Neoplasms"[Mesh])) OR (Adenoma*[Title/Abstract])) OR (Polyp*[Title/Abstract])) OR (Neoplasm*[Title/Abstract])) OR (Neoplasia*[Title/Abstract])) OR (Serrated) |
|  | #2 | ((((((((("Colon"[Mesh]) OR ("Rectum"[Mesh])) OR ("Intestines"[Mesh])) OR (colon*[Title/Abstract])) OR (Rectum*[Title/Abstract])) OR (Colorectal[Title/Abstract])) OR (Colorectum[Title/Abstract])) OR (Bowel[Title/Abstract])) OR (Intestine*[Title/Abstract])) OR (Intestinal[Title/Abstract]) |
|  | #3 | ((((((((((((((((((((("Artificial Intelligence"[Mesh]) OR ("Diagnosis, Computer-Assisted"[Mesh])) OR (Artificial Intelligence[Title/Abstract])) OR (AI[Title/Abstract])) OR (CAD[Title/Abstract])) OR (CADe[Title/Abstract])) OR (CADx[Title/Abstract])) OR (Computer Aided Detection)) OR (Computer Assisted Detection)) OR (Computer Aided Diagnosis)) OR (Computer Assisted System*)) OR (EndoScreener)) OR (EndoAngel)) OR (GI Genius)) OR (GI-Genius)) OR (EndoVigilant)) OR (CAD EYE)) OR (CAD-EYE)) OR (Endo-Aid)) OR (EndoBRAIN)) OR (Eagle-Eye)) OR (SKOUT) |
|  | #4 | (((((((((("Randomized Controlled Trial" [Publication Type]) OR ("Clinical Trials as Topic"[Mesh])) OR ("Controlled Clinical Trial" [Publication Type])) OR ("Pragmatic Clinical Trial" [Publication Type])) OR (trial*[Title/Abstract])) OR (random*[Title/Abstract])) OR (placebo[Title/Abstract])) OR ((controlled) and (study or design or trial))) OR (intermethod comparison)) OR ((singl* or doubl* or trebl* or tripl*) and (blind* or mask*))) OR ((cross over) or crossover or cross-over) |
|  | #5 | #1 AND #2 AND #3 AND #4 |
| **Embase** | #1 | 'adenoma'/exp OR 'polyp'/exp OR 'neoplasm'/exp OR adenoma*:ab,ti OR polyp*:ab,ti OR neoplasm*:ab,ti OR neoplasia*:ab,ti OR serrated |
|  | #2 | 'colon'/exp OR 'rectum'/exp OR 'intestine'/exp OR colon*:ab,ti OR rectum*:ab,ti OR colorectal:ab,ti OR colorectum:ab,ti OR bowel:ab,ti OR intestine*:ab,ti OR intestinal:ab,ti |
|  | #3 | 'artificial intelligence'/exp OR 'computer aided detection'/exp OR 'artificial intelligence':ab,ti OR ai:ab,ti OR cad:ab,ti OR cade:ab,ti OR cadx:ab,ti OR 'computer aided detection' OR 'computer assisted detection' OR 'computer aided diagnosis' OR 'computer assisted system*' OR endoscreener OR endoangel OR 'gi genius' OR 'cad eye' OR 'endo aid' OR endobrain OR 'eagle eye' OR skout OR endovigilant |
|  | #4 | 'randomized controlled trial'/exp OR 'clinical trial'/exp OR trial*:ab,ti OR (controlled:ab,ti AND (study:ab,ti OR design:ab,ti OR trial:ab,ti)) OR random* OR 'intermethod comparison' OR placebo:ab,ti OR ((singl* OR doubl* OR trebl* OR tripl*) AND (blind* OR mask*)) OR ('cross over' OR crossover) |
|  | #5 | #1 AND #2 AND #3 AND #4 |
| **MEDLINE** | #1 | Adenoma/ or Polyps/ or Neoplasms/ or Adenoma*.ab,kw,ti. or Polyp*.ab,kw,ti. or Neoplasm*.ab,kw,ti. or Neoplasia*.ab,kw,ti. or Serrated.af. |
|  | #2 | Colon/ or Rectum/ or Intestines/ or colon*.ab,kw,ti. or Rectum*.ab,kw,ti. or Colorectal.ab,kw,ti. or Colorectum.ab,kw,ti. or Bowel.ab,kw,ti. or Intestine*.ab,kw,ti. or Intestinal.ab,kw,ti. |
|  | #3 | Artificial Intelligence/ or Diagnosis, Computer-Assisted/ or Artificial Intelligence.ab,kw,ti. or AI.ab,kw,ti. or CAD.ab,kw,ti. or CADe.ab,kw,ti. or CADx.ab,kw,ti. or Computer Aided Detection.af. or Computer Assisted Detection.af. or Computer Aided Diagnosis.af. or Computer Assisted System*.af. or EndoScreener.af. or EndoAngel.af. or GI Genius.af. or CAD EYE.af. or Endo-Aid.af. or EndoBRAIN.af. or Eagle-Eye.af. or SKOUT.af. or EndoVigilant.af. |
|  | #4 | randomized controlled trial.pt. or controlled clinical trial.pt. or pragmatic clinical trial.pt. or Clinical Trial/ or trial*.ab,kw,ti. or (controlled and (study or design or trial)).af. or random*.ab,kw,ti. or placebo.ab,kw,ti. or intermethod comparison.af. or ((singl* or doubl* or trebl* or tripl*) and (blind* or mask*)).af. or cross over.af. or crossover.af. |
|  | #5 | #1 AND #2 AND #3 AND #4 |
| **Scopus** | #1 | (TITLE-ABS-KEY (adenoma* OR polyp* OR neoplasm* OR neoplasia*) OR ALL (serrated)) AND TITLE-ABS-KEY (colon* OR Rectum* OR Colorectal OR Colorectum OR Bowel OR Intestine* OR Intestinal) AND (TITLE-ABS-KEY (Artificial Intelligence OR AI OR CAD OR CADe OR CADx) OR ALL (“Computer Aided Detection” OR “Computer Assisted Detection” OR “Computer Aided Diagnosis” OR “Computer Assisted System*” OR EndoScreener OR EndoAngel OR “GI Genius” OR “CAD EYE” OR Endo-Aid OR EndoBRAIN OR Eagle-Eye OR SKOUT OR EndoVigilant)) AND (ALL (“Randomized controlled trial” OR (controlled and (study or design or trial)) OR “intermethod comparison” OR ((singl* or doubl* or trebl* or tripl*) and (blind* or mask*)) OR “cross over” OR crossover) OR TITLE-ABS-KEY (random* OR placebo)) |
| **Web of Science** | #1 | (TS=(Adenoma OR Neoplasms OR Polyps) OR TI=(Neoplasm* OR Polyp* OR Adenoma* OR Neoplasia*) OR AB=(Neoplasm* OR Polyp* OR Adenoma* OR Neoplasia*) OR ALL=Serrated) AND (TS=(Colon OR Rectum OR Intestines) OR TI=(colon* OR Rectum* OR Colorectal OR Colorectum OR Bowel OR Intestine* OR Intestinal) OR AB=(colon* OR Rectum* OR Colorectal OR Colorectum OR Bowel OR Intestine* OR Intestinal)) AND (TS=(Artificial Intelligence OR Diagnosis, Computer-Assisted) OR TI=(Artificial Intelligence OR AI OR CAD OR CADe OR CADx) OR AB=(Artificial Intelligence OR AI OR CAD OR CADe OR CADx) OR ALL=(Computer Aided Detection OR Computer Assisted Detection OR Computer Aided Diagnosis OR Computer Assisted System* OR EndoScreener OR EndoAngel OR GI Genius OR GI-Genius OR CAD EYE OR CAD-EYE OR Endo-Aid OR EndoBRAIN OR Eagle-Eye OR SKOUT OR EndoVigilant)) AND (TS=trial* OR ALL=(Randomized controlled trial OR (controlled and (study or design or trial)) OR intermethod comparison OR ((singl* or doubl* or trebl* or tripl*) and (blind* or mask*)) OR cross over OR crossover OR cross-over) OR TI=(random* OR placebo) OR AB=(random* OR placebo)) |
| **CENTRAL** | #1 | MeSH descriptor: [Adenoma] or MeSH descriptor: [Polyps] or MeSH descriptor: [Neoplasms] explode all trees or (Adenoma*):ti,ab,kw or (Polyp*):ti,ab,kw or (Neoplasm*):ti,ab,kw or (Neoplasia*):ti,ab,kw or (Serrated) |
|  | #2 | MeSH descriptor: [Colon] or MeSH descriptor: [Rectum] or MeSH descriptor: [Intestines] or (colon*):ti,ab,kw or (Rectum*):ti,ab,kw or (Colorectal):ti,ab,kw or (Colorectum):ti,ab,kw or (Bowel):ti,ab,kw or (Intestine*):ti,ab,kw or (Intestinal):ti,ab,kw |
|  | #3 | MeSH descriptor: [Artificial Intelligence] or (Artificial Intelligence):ti,ab,kw or (AI):ti,ab,kw or (CAD):ti,ab,kw or (Computer Aided Detection) or (Computer Assisted Detection) or (Computer Aided Diagnosis) or (Computer Assisted System*) or EndoScreener or EndoAngel or (GI Genius) or (CAD EYE) or Endo-Aid or EndoBRAIN or Eagle-Eye or SKOUT or EndoVigilant |
|  | #4 | #1 AND #2 AND #3 |

**Table S2.** Details of the Network Meta-analysis Models.

| **Outcome** | **linearModel** | **n.adapt** | **n.iter** |
| --- | --- | --- | --- |
| **ADR-mITT** | Random | 40,000 | 70,000 |
| **ADR-ITT** | Random | 50,000 | 80,000 |
| **APC** | Random | 20,000 | 50,000 |
| **DADR** | Random | 20,000 | 50,000 |
| **AADR** | Random | 20,000 | 50,000 |
| **SDR** | Random | 20,000 | 50,000 |
| **WT** | Random | 20,000 | 50,000 |

**Table S3. The heterogeneity of the studies on ADR based on modified intention-to-treat.**

| **Comparison** | **Studies (n)** | ***p*** | ***I*² (%)** |
| --- | --- | --- | --- |
| **CAD EYE vs CC** | 4 | 0.6558 | 0 |
| **DEEP² vs CC** | 1 | - | - |
| **Eagle-Eye vs CC** | 1 | - | - |
| **ENDO-AID vs CC** | 3 | 0.9032 | 0 |
| **EndoScreener vs CC** | 1 | - | - |
| **EndoVigilant vs CC** | 1 | - | - |
| **GI-Genius vs CC** | 3 | 0.5225 | 0 |
| **EndoAngel vs CC** | 1 | - | - |
| **Overall Network** | 15 | 0.8739 | 0 |

**Table S4. The heterogeneity of the studies on ADR based on intention-to-treat.**

| **Comparison** | **Studies (n)** | ***p*** | ***I*² (%)** |
| --- | --- | --- | --- |
| **CAD EYE vs CC** | 1 | - | - |
| **Eagle-Eye vs CC** | 1 | - | - |
| **ENDO-AID vs CC** | 2 | 0.8998 | 0 |
| **EndoScreener vs CC** | 2 | 0.4055 | 0 |
| **EndoVigilant vs CC** | 1 | - | - |
| **GI-Genius vs CC** | 1 | - | - |
| **AQCS vs CC** | 1 | - | - |
| **Overall Network** | 9 | 0.7019 | 0 |

**Table S5.** The heterogeneity of the studies on APC.

| **Comparison** | **Studies (n)** | ***p*** | ***I*²** **(%)** |
| --- | --- | --- | --- |
| **CAD EYE vs CC** | 4 | 0.0361 | 64.9 |
| **Eagle-Eye vs CC** | 1 | - | - |
| **ENDO-AID vs CC** | 3 | 0.3830 | 0 |
| **EndoScreener vs CC** | 1 | - | - |
| **EndoVigilant vs CC** | 1 | - | - |
| **GI-Genius vs CC** | 3 | 0.8806 | 0 |
| **EndoAngel vs CC** | 1 | - | - |
| **Overall Network** | 14 | 0.1518 | 26 |

**Table S6. The heterogeneity of the studies on DADR based on intention-to-treat.**

| **Comparison** | **Studies (n)** | ***p*** | ***I*² (%)** |
| --- | --- | --- | --- |
| **CAD EYE vs CC** | 1 | - | - |
| **DEEP² vs CC** | 1 | - | - |
| **Eagle-Eye vs CC** | 1 | -- |  |
| **ENDO-AID vs CC** | 2 | 0.4500 | 0 |
| **GI-Genius vs CC** | 1 | - | - |
| **EndoAngel vs CC** | 1 | - | - |
| **Overall Network** | 7 | 0.4499 | 0 |

**Table S7. The heterogeneity of the studies on AADR.**

| **Comparison** | **Studies (n)** | ***p*** | ***I*² (%)** |
| --- | --- | --- | --- |
| **CAD EYE vs CC** | 1 | - | - |
| **DEEP² vs CC** | 1 | - | - |
| **Eagle-Eye vs CC** | 1 | - | - |
| **ENDO-AID vs CC** | 3 | 0.4110 | 0 |
| **GI-Genius vs CC** | 2 | 0.1502 | 51.7 |
| **EndoAngel vs CC** | 1 | - | - |
| **Overall Network** | 9 | 0.2784 | 20.7 |

**Table S8. The heterogeneity of the studies on SDR.**

| **Comparison** | **Studies (n)** | ***p*** | ***I*² (%)** |
| --- | --- | --- | --- |
| **CAD EYE vs CC** | 2 | 0.1568 | 50.1 |
| **Eagle-Eye vs CC** | 1 | - | - |
| **ENDO-AID vs CC** | 2 | 0.9632 | 0 |
| **EndoVigilant vs CC** | 1 | - | - |
| **EndoAngel vs CC** | 1 | - | - |
| **Overall Network** | 7 | 0.3668 | 25 |

**Table S9. The heterogeneity of the studies on WT.**

| **Comparison** | **Studies (n)** | ***p*** | ***I*² (%)** |
| --- | --- | --- | --- |
| **CAD EYE vs CC** | 3 | 0.6394 | 0 |
| **DEEP² vs CC** | 1 | - | - |
| **ENDO-AID vs CC** | 3 | 0.1359 | 49.9 |
| **EndoScreener vs CC** | 1 | - | - |
| **EndoVigilant vs CC** | 1 | - | - |
| **GI-Genius vs CC** | 1 | - | - |
| **EndoAngel vs CC** | 1 | - | - |
| **Overall Network** | 11 | 0.2992 | 25 |

**Table S10.** **Effect estimates for CADe system compared with conventional colonoscopy/Risk Ratio [95% CI].**

| **CADe** **systems** | **Without extended functions** | **With and without extended functions** |
| --- | --- | --- |
| **CAD EYE** | 1.06 [0.96, 1.17] | - |
| **DEEP^2^** | 1.37 [1.09, 1.71] | - |
| **Eagle-Eye** | 1.19 [1.07, 1.31] | - |
| **ENDO-AID** | **1.26 [1.16, 1.38]** | **1.28 [1.17, 1.39]** |
| **EndoScreener** | 1.31 [0.96, 1.79] | - |
| **EndoVigilant** | 0.97 [0.80, 1.16] | - |
| **GI-Genius** | 1.05 [1.00, 1.10] | - |
| **EndoAngel** | **1.44 [1.00, 2.08]** | **1.68 [1.20, 2.35]** |
| **Total** | **1.14 [1.07, 1.21]** | **1.16 [1.09, 1.24]** |

**Table S11. GRADE assessment results for ADR based on modified intention-to-treat.**

| **Quality assessment** | | | | | | | **No of patients** | | **Effect** | | **Quality** |
| --- | --- | --- | --- | --- | --- | --- | --- | --- | --- | --- | --- |
|  |  |  |  |  |  |  |  |  |  |  |  |
| **No of studies** | **Design** | **Risk of bias** | **Inconsistency** | **Indirectness** | **Imprecision** | **Other considerations** | **Intervention** | **Control** | **Relative (95% CrI)** | **Absolute** |  |
| **CAD EYE vs CC** | | | | | | | | | | | |
| 4 | randomised trials | serious^1^ | no serious inconsistency | no serious indirectness | serious^2^ | none | 364/721 (50.5%) | 348/737 (47.2%) | RR 1.06 (0.94 to 1.19) | 28 more per 1000 (from 28 fewer to 90 more) | ⊕⊕OO LOW |
|  |  |  |  |  |  |  |  | 0% |  | - |  |
| **DEEP² vs CC** | | | | | | | | | | | |
| 1 | randomised trials | serious^1^ | no serious inconsistency | no serious indirectness | no serious imprecision | none | 122/330 (37%) | 93/344 (27%) | RR 1.37 (1.07 to 1.76) | 100 more per 1000 (from 19 more to 205 more) | ⊕⊕⊕O MODERATE |
|  |  |  |  |  |  |  |  | 0% |  | - |  |
| **Eagle-Eye vs CC** | | | | | | | | | | | |
| 1 | randomised trials | serious^1^ | no serious inconsistency | no serious indirectness | no serious imprecision | none | 492/1238 (39.7%) | 432/1289 (33.5%) | RR 1.19 (1.02 to 1.38) | 64 more per 1000 (from 7 more to 127 more) | ⊕⊕⊕O MODERATE |
|  |  |  |  |  |  |  |  | 0% |  | - |  |
| **ENDO-AID vs CC** | | | | | | | | | | | |
| 3 | randomised trials | serious^1^ | no serious inconsistency | no serious indirectness | no serious imprecision | none | 600/1119 (53.6%) | 474/1117 (42.4%) | RR 1.27 (1.13 to 1.41) | 115 more per 1000 (from 55 more to 174 more) | ⊕⊕⊕O MODERATE |
|  |  |  |  |  |  |  |  | 0% |  | - |  |
| **EndoScreener vs CC** | | | | | | | | | | | |
| 1 | randomised trials | serious^1^ | no serious inconsistency | no serious indirectness | no serious imprecision | none | 64/184 (34.8%) | 49/185 (26.5%) | RR 1.31 (0.94 to 1.83) | 82 more per 1000 (from 16 fewer to 220 more) | ⊕⊕⊕O MODERATE |
|  |  |  |  |  |  |  |  | 0% |  | - |  |
| **EndoVigilant vs CC** | | | | | | | | | | | |
| 1 | randomised trials | serious^1^ | no serious inconsistency | no serious indirectness | no serious imprecision | none | 139/387 (35.9%) | 14/382 (3.7%) | RR 0.97 (0.78 to 1.20) | 1 fewer per 1000 (from 8 fewer to 7 more) | ⊕⊕⊕O MODERATE |
|  |  |  |  |  |  |  |  | 0% |  | - |  |
| **GI-Genius vs CC** | | | | | | | | | | | |
| 3 | randomised trials | serious^3^ | no serious inconsistency | no serious indirectness | no serious imprecision | none | 1481/2727 (54.3%) | 1401/2722 (51.5%) | RR 1.05 (0.97 to 1.15) | 26 more per 1000 (from 15 fewer to 77 more) | ⊕⊕⊕O MODERATE |
|  |  |  |  |  |  |  |  | 0% |  | - |  |
| **EndoAngel vs CC** | | | | | | | | | | | |
| 1 | randomised trials | no serious risk of bias | no serious inconsistency | no serious indirectness | no serious imprecision | none | 57/268 (21.3%) | 40/271 (14.8%) | RR 1.45 (0.98 to 2.15) | 66 more per 1000 (from 3 fewer to 170 more) | ⊕⊕⊕⊕ HIGH |
|  |  |  |  |  |  |  |  | 0% |  | - |  |
| **EndoAngel vs DEEP²** | | | | | | | | | | | |
| 0 | randomised trials | serious^4^ | no serious inconsistency | serious^5^ | no serious imprecision | none | 57/268 (21.3%) | 122/330 (37%) | RR 1.06 (0.67 to 1.68) | 22 more per 1000 (from 122 fewer to 251 more) | ⊕⊕OO LOW |
|  |  |  |  |  |  |  |  | 0% |  | - |  |
| **EndoAngel vs EndoScreener** | | | | | | | | | | | |
| 0 | randomised trials | serious^4^ | no serious inconsistency | serious^6^ | no serious imprecision | none | 57/268 (21.3%) | 64/184 (34.8%) | RR 1.10 (0.66 to 1.86) | 35 more per 1000 (from 118 fewer to 299 more) | ⊕⊕OO LOW |
|  |  |  |  |  |  |  |  | 0% |  | - |  |
| **DEEP² vs EndoScreener** | | | | | | | | | | | |
| 0 | randomised trials | serious^1^ | no serious inconsistency | serious^7^ | no serious imprecision | none | 122/330 (37%) | 64/184 (34.8%) | RR 1.04 (0.69 to 1.58) | 14 more per 1000 (from 108 fewer to 202 more) | ⊕⊕OO LOW |
|  |  |  |  |  |  |  |  | 0% |  | - |  |

^1^ All studies were some-concern risk of bias in reporting bias.
^2^ Two of the four studies have small sample sizes (<200).
^3^ One of the three studies was some-concern risk of bias in reporting bias.
^4^ One of the two studies was some-concern risk of bias in reporting bias.
^5^ The comparison is based on the results of EndoAngel versus CC and DEEP²versus CC.
^6^ The comparison is based on the results of EndoAngel versus CC and EndoScreener versus CC.

^7^ The comparison is based on the results of DEEP²versus CC and EndoScreener versus CC.

**Table S12.** GRADE assessment results for ADR based on intention-to-treat.

| **Quality assessment** | | | | | | | **No of patients** | | **Effect** | | **Quality** |
| --- | --- | --- | --- | --- | --- | --- | --- | --- | --- | --- | --- |
|  |  |  |  |  |  |  |  |  |  |  |  |
| **No of studies** | **Design** | **Risk of bias** | **Inconsistency** | **Indirectness** | **Imprecision** | **Other considerations** | **Intervention** | **Control** | **Relative (95% CrI)** | **Absolute** |  |
| **CAD EYE vs CC** | | | | | | | | | | | |
| 1 | randomised trials | serious^1^ | no serious inconsistency | no serious indirectness | no serious imprecision | none | 123/207 (59.4%) | 99/208 (47.6%) | RR 1.25 (0.87 to 1.79) | 119 more per 1000 (from 62 fewer to 376 more) | ⊕⊕⊕O MODERATE |
|  |  |  |  |  |  |  |  | 0% |  | - |  |
| **Eagle-Eye vs CC** | | | | | | | | | | | |
| 1 | randomised trials | serious^1^ | no serious inconsistency | no serious indirectness | no serious imprecision | none | 606/1519 (39.9%) | 499/1540 (32.4%) | RR 1.23 (0.88 to 1.71) | 75 more per 1000 (from 39 fewer to 230 more) | ⊕⊕⊕O MODERATE |
|  |  |  |  |  |  |  |  | 0% |  | - |  |
| **ENDO-AID vs CC** | | | | | | | | | | | |
| 2 | randomised trials | serious^1^ | no serious inconsistency | no serious indirectness | no serious imprecision | none | 404/796 (50.8%) | 327/802 (40.8%) | RR 1.25 (0.98 to 1.60) | 102 more per 1000 (from 8 fewer to 245 more) | ⊕⊕⊕O MODERATE |
|  |  |  |  |  |  |  |  | 0% |  | - |  |
| **EndoScreener vs CC** | | | | | | | | | | | |
| 2 | randomised trials | serious^1^ | no serious inconsistency | no serious indirectness | no serious imprecision | none | 279/877 (31.8%) | 217/875 (24.8%) | RR 1.28 (1.00 to 1.69) | 69 more per 1000 (from 0 more to 171 more) | ⊕⊕⊕O MODERATE |
|  |  |  |  |  |  |  |  | 0% |  | - |  |
| **EndoVigilant vs CC** | | | | | | | | | | | |
| 1 | randomised trials | no serious risk of bias | no serious inconsistency | no serious indirectness | no serious imprecision | none | 85/124 (68.5%) | 96/120 (80%) | RR 0.86 (0.61 to 1.21) | 112 fewer per 1000 (from 312 fewer to 168 more) | ⊕⊕⊕⊕ HIGH |
|  |  |  |  |  |  |  |  | 0% |  | - |  |
| **GI-Genius vs CC** | | | | | | | | | | | |
| 1 | randomised trials | serious^1^ | no serious inconsistency | no serious indirectness | no serious imprecision | none | 176/330 (53.3%) | 147/330 (44.5%) | RR 1.20 (0.85 to 1.70) | 89 more per 1000 (from 67 fewer to 312 more) | ⊕⊕⊕O MODERATE |
|  |  |  |  |  |  |  |  | 0% |  | - |  |
| **AQCS vs CC** | | | | | | | | | | | |
| 1 | randomised trials | no serious risk of bias | no serious inconsistency | no serious indirectness | no serious imprecision | none | 205/627 (32.7%) | 142/627 (22.6%) | RR 1.44 (1.02 to 2.06) | 100 more per 1000 (from 5 more to 240 more) | ⊕⊕⊕⊕ HIGH |
|  |  |  |  |  |  |  |  | 0% |  | - |  |
| **AQCS vs EndoScreener** | | | | | | | | | | | |
| 0 | randomised trials | serious^2^ | no serious inconsistency | serious^3^ | no serious imprecision | none | 205/627 (32.7%) | 279/877 (31.8%) | RR 1.12 (0.72 to 1.74) | 38 more per 1000 (from 89 fewer to 235 more) | ⊕⊕OO LOW |
|  |  |  |  |  |  |  |  | 0% |  | - |  |
| **AQCS vs CAD EYE** | | | | | | | | | | | |
| 0 | randomised trials | serious^4^ | no serious inconsistency | serious^5^ | no serious imprecision | none | 205/627 (32.7%) | 123/207 (59.4%) | RR 1.15 (0.70 to 1.91) | 89 more per 1000 (from 178 fewer to 541 more) | ⊕⊕OO LOW |
|  |  |  |  |  |  |  |  | 0% |  | - |  |
| **EndoScreener vs CAD EYE** | | | | | | | | | | | |
| 0 | randomised trials | serious^1^ | no serious inconsistency | serious^6^ | no serious imprecision | none | 279/877 (31.8%) | 123/207 (59.4%) | RR 1.03 (0.67 to 1.61) | 18 more per 1000 (from 196 fewer to 362 more) | ⊕⊕OO LOW |
|  |  |  |  |  |  |  |  | 0% |  | - |  |

^1^ All studies were some-concern risk of bias in reporting bias.
^2^ Two of the three studies were some-concern risk of bias in reporting bias.
^3^ The comparison is based on the results of AQCS versus CC and EndoScreener versus CC.
^4^ One of the two studies was some-concern risk of bias in reporting bias.
^5^ The comparison is based on the results of AQCS versus CC and CAD EYE versus CC.
^6^ The comparison is based on the results of EndoScreener versus CC and CAD EYE versus CC.

**Table S13**. Key features of CADe systems.

| **System** | **Developer** | **Architecture** | **Training Data** | **Sensitivity** | **Latency** | **Alert Mechanism** | **Special Features** |
| --- | --- | --- | --- | --- | --- | --- | --- |
| DEEP^2^ | Fujifilm/  Google  /Verily | DNN | >3,000 videos | Per-polyp 97.1% | - | Box + thumbnail (5s) + audio (once) | Anti-alarm fatigue |
| EndoScreener | Wision AI, China | SegNet | - | Per-polyp 100%; per-image 91.64–94.38% | ~20 ms | Box + audio | Dual/single monitor; direct video display |
| ENDO-AID | Olympus, Japan | CNN | 120,000 images | Per-polyp 97.5–100% | <50 ms | Flag + picture-in-picture (normal mode); box (target mode) | Two sensitivity modes |
| EndoAngel | EndoAngel, China | YOLO v3 | 20,000 images | Per-frame 97.31%; per-polyp 95.8–97.3% | - | Box + audio | Full-process QC; skip cecum button |
| AQCS | Qilu Hospital  /Medicon | 5 DCNN models | >15,000 images | Per-polyp 96.04% | 25 fps | Box + audio | Timing; stability; bowel prep alerts |
| CAD EYE | Fujifilm, Japan | - | - | 94.5% (WLI) | 200–300 ms | Box + audio | Integrated CADe/CADx; button activation |

**Figure S1.** Assessment of the model convergence for ADR based on modified intention-to-treat (Brooks-Gelman-Rubin plots).

**Figure S2.** The networks of included randomized controlled trials. Network diagram for ADR-mITT (A), ADR-ITT (B), APC (C), DADR (D), AADR (E), SDR (F), and WT (G).

**Figure S3.** Risk of bias summary.

**Figure S4.** Comprehensive estimate for the comparison of outcomes in the network meta-analysis.

CC, conventional colonoscopy; ADR-mITT, adenoma detection rate based on modified intention-to-treat; ADR-ITT, adenoma detection rate based on intention-to-treat; APC, adenoma per colonoscopy; DADR, diminutive adenoma detection rate; AADR, advanced adenoma detection rate; SDR, sessile serrated lesion detection rate; WT, withdrawal time.

(A) Comprehensive estimate for the comparison of ADR-mITT, RR (95% Crl).

(B) Comprehensive estimate for the comparison of ADR-ITT, RR (95% Crl).

(C) Comprehensive estimate for APC, MD (95% Crl).

(D) Comprehensive estimate for DADR, RR (95% Crl).

(E) Comprehensive estimate for AADR, RR (95% Crl).

(F) Comprehensive estimate for SDR, RR (95% Crl).

(G) Comprehensive estimate for WT, MD (95% Crl).

**Figure S5.** Egger’s test for the included studies involving ADR based on modified intention-to-treat.

**Figure S6.** Assessment of the transitivity.

A: Population characteristics of age; B: Population characteristics of country; C: Indication for colonoscopy; D: Endoscopist experience; E: Baseline ADR; F: Bowel preparation quality; G: Single-center and multicenter design.
